# Cerebrovascular Disease and Perioperative Neurologic Vulnerability: A Prospective Cohort Study

**DOI:** 10.3389/fneur.2019.00560

**Published:** 2019-05-28

**Authors:** Phillip E. Vlisides, Bryan Kunkler, Aleda Thompson, Mackenzie Zierau, Remy Lobo, Mary O. Strasser, Michael J. Cantley, Amy McKinney, Allen D. Everett, George A. Mashour, Paul Picton

**Affiliations:** ^1^Department of Anesthesiology, University of Michigan Medical School, Ann Arbor, MI, United States; ^2^Center for Consciousness Science, University of Michigan Medical School, Ann Arbor, MI, United States; ^3^Department of Radiology, University of Michigan Medical School, Ann Arbor, MI, United States; ^4^Pediatric Proteome Center, Johns Hopkins University School of Medicine, Baltimore, MD, United States

**Keywords:** biomarkers, cerebrovascular disease, cognitive dysfunction, hypoxia-ischemia, perioperative care, stroke

## Abstract

**Background:** Stroke is a devastating perioperative complication without effective methods for prevention or diagnosis. The objective of this study was to analyze evidence-based strategies for detecting cerebrovascular vulnerability and injury in a high-risk cohort of non-cardiac surgery patients.

**Methods:** This was a single-center, prospective cohort study. Fifty patients undergoing non-cardiac surgery were recruited −25 with known cerebrovascular disease and 25 matched controls. Neurologic vulnerability was measured with intraoperative cerebral oximetry as the primary outcome. Perioperative neurocognitive testing and serum biomarker analysis (S-100β, neuron specific enolase, glial fibrillary acid protein, and matrix metalloproteinase-9) were measured as secondary outcomes.

**Results:** Cerebral desaturation events (an oximetry decrease ≥20% from baseline or <50% absolute value for ≥3 min) occurred in 7/24 (29%) cerebrovascular disease patients and 2/24 (8.3%) controls (relative risk 3.5, 95% CI 0.81–15.2; *P* = 0.094). Cognitive function trends were similar in both groups, though overall scores (range: 1,500–7,197) were ~1 standard deviation lower in cerebrovascular patients across the entire perioperative period (−1,049 [95% CI −1,662, −436], *P* < 0.001). No significant serum biomarker differences were found between groups over time. One control patient experienced intraoperative hypoxic-ischemic injury, but no robust biomarker or oximetry changes were observed.

**Conclusions:** Cerebrovascular disease patients did not demonstrate dramatic differences in cerebral oximetry, cognitive trajectory, or molecular biomarkers compared to controls. Moreover, a catastrophic hypoxic-ischemic event was neither predicted nor detected by any strategy tested. These findings support the need for novel research into cerebrovascular risk and vulnerability.

## Introduction

Perioperative stroke results in an 8-fold increase in mortality (1), functional disability (2), and high likelihood of discharge to long-term care facilities (2, 3). Although generally a rare event, perioperative stroke risk approaches 2% in high-risk patients (1), and clinically silent (i.e., covert) stroke may occur with a startling 10% incidence after non-cardiac surgery (4). Recognition and diagnosis of postoperative stroke is often delayed, and thrombolysis is rarely considered (2, 3). Furthermore, mortality rates have been reported as high as 84% in non-cardiac surgery patients (2). Despite these grave outcomes associated with perioperative stroke, no formal guidelines exist for prevention and management. The Society for Neuroscience in Anesthesiology and Critical Care has released a consensus statement, but many of the recommendations are based on low-quality evidence and expert opinion (5).

Evidence-based methods to detect cerebrovascular vulnerability and injury are desperately needed to mitigate stroke risk in surgical patients. Those with cerebrovascular disease history (e.g., stroke, transient ischemic attack, carotid artery stenosis) have a particularly high risk of perioperative stroke (1, 6–9), though there are no standard methods for monitoring cerebrovascular function perioperatively for non-cardiac surgery. One candidate method for monitoring cerebrovascular function is near-infrared spectroscopy-based cerebral oximetry. Cerebral oximetry has been purported to estimate balance between cerebral oxygen supply and demand (10), and specific desaturation thresholds have correlated with clinical and neurophysiologic signs of hypoxic-ischemic injury (11–16). Furthermore, given the autoregulation impairment present with cerebrovascular disease (17, 18), cerebral oximetry may serve as a useful tool for monitoring intraoperative cerebrovascular function. Additionally, cognitive dysfunction and decline and candidate brain injury biomarkers (e.g., S-100β, glial fibrillary acid protein) may also reflect ongoing cerebral hypoxic-ischemic events in non-surgical settings (19, 20); studying such cognitive function trends and molecular biomarkers may thus be useful for detecting cerebrovascular vulnerability and injury perioperatively.

The primary aim of this study is to determine whether pre-defined intraoperative cerebral desaturation events commonly occur in a high-risk group, i.e., those with prior cerebrovascular disease history. Secondary aims are to characterize perioperative molecular biomarkers and neurocognitive profiles in high-risk, cerebrovascular disease patients and matched controls.

## Materials and Methods

This was a single-center, prospective cohort study conducted at Michigan Medicine (Ann Arbor, MI USA), and it was approved by the University of Michigan Medical School Institutional Review Board (HUM00106530, approved 11/2/2016). Screening and recruitment procedures were performed by various study team members (PEV, BK, AT, MZ, MOS, MJC, AM) under direction supervision of the study PI (PEV). Patients were recruited between May 2016 and September 2017 at preoperative clinic and check-in locations. Written informed consent was obtained from all participants prior to enrollment. This study fulfills STROBE criteria outlined for observational studies (Supplementary Table 1) (21).

### Study Population

Adult (≥18 years old) surgical patients presenting for non-cardiac, non-neurologic, and non-major vascular surgery were included. Surgical procedures were selected that did not involve direct manipulation of brain parenchyma or vascular blood flow to or from the brain itself, as our primary aim was to detect signs of vulnerability and injury based on the medical comorbidity of cerebrovascular disease. Surgical patients with a broad cerebrovascular disease history were included. Inclusion criteria were prior stroke, history of transient ischemic attack, or history of documented carotid artery stenosis. Prior stroke and transient ischemic attack have both been identified as independent risk factors for perioperative stroke (1, 6, 8), and surgical patients with carotid artery stenosis demonstrate an elevated incidence of postoperative stroke (2–3.6%) (7, 9) compared to general population estimates (0.1–0.7%) (1, 8). Furthermore, those with carotid artery stenosis may demonstrate impaired cerebral autoregulation (17). Thus, we purposefully kept cerebrovascular disease inclusion criteria broad to include all patient subtypes that could conceivably demonstrate increased stroke vulnerability perioperatively. In this study, carotid artery stenosis was defined as having a diagnosis in the medical record and either prior or pending referral for medical or surgical evaluation (e.g., antiplatelet therapy, endarterectomy). There was otherwise no quantitative cutoff for inclusion.

Exclusion criteria included the following: emergency surgery, patients in respiratory failure (i.e., requiring invasive or non-invasive assisted ventilation), patients with severe pre-existing cognitive impairment (precluding capacity for obtaining informed consent), pregnancy, or patients unable to speak English. Control cases were matched (1:1) based on sex, age (within 10 years of age), and surgical subtype.

### Anesthetic and Perioperative Procedures

After enrollment, study team members collected baseline demographic information, past medical history, and extensive details regarding cerebrovascular disease history. For patients with prior stroke, clinical and chart review was conducted to determine the following information, when available: when the stroke occurred, territory affected, cause of stroke, and all treatments and interventions provided (Supplementary Table 2). Current level of disability was also assessed using the Modified Rankin Scale as was the existence of pre-existing neurologic deficits using the modified National Institutes of Health Stroke Scale. Study team members performing these assessments were certified through the American Heart Association/American Stroke Association. Lastly, preoperative baseline cognitive function was assessed using our iPad test battery as described below in further detail.

In the preoperative holding area, study participants had near-infrared spectroscopy sensors (INVOS 5100C monitoring system Medtronic, Minneapolis, MN, USA) applied to each side of their forehead for continuous monitoring of regional cerebral oxygenation. Baseline values were recorded prior to any premedication with the patient sitting upright and breathing room air for a minimum of 5 min. Blood samples were also drawn preoperatively for baseline serum biomarker analysis.

Once in the operating room, patients were monitored according to American Society of Anesthesiologists standards. After induction of general anesthesia and endotracheal intubation, ventilation proceeded with tidal volumes of 6 to 8 mL/kg to target end-tidal carbon dioxide levels of 35–40 mmHg. The inspired oxygen fraction was set to 50%, and nitrous oxide was avoided. These ventilator parameters were prospectively maintained to minimize physiologic confounding of oximetry values. Intraoperative management was otherwise left to the discretion of surgical and anesthesia teams. Physiologic data from each case were electronically imported from our anesthesia information management system (Centricity; General Electric Healthcare, Waukesha, WI USA) for further analysis.

Postoperative visits occurred for the first 3 days after surgery, and all visits occurred between 8:15 am and 1:45 pm. During each study visit, modified National Institutes of Health Stroke Scale was calculated and cognitive function testing was performed (details described below). Serum biomarkers were also drawn during scheduled phlebotomy morning rounds (3:00 am−10:00 am) on each of the first three postoperative days. Study operations concluded at the end of the third postoperative study visit.

### Study Measures

The primary outcome was incidence of sustained (≥3 min) intraoperative cerebral desaturation—predefined as a ≥20% relative decrease from baseline or absolute value <50%. We chose these specific thresholds as they have correlated with neurophysiologic (11–13) or clinical (12, 14–16) signs of cerebral ischemia. Secondary outcomes included postoperative changes in cognitive function and the following serum biomarkers, based on their reported association with cerebral ischemic injury: S-100β (ng/L) (19), glial fibrillary acid protein (ng/mL) (19, 22), neuron specific enolase (ng/mL) (23), and matrix metalloproteinase-9 (ng/mL) (24).

### Cerebral Oximetry Analysis

Cerebral oximetry was measured using the INVOS 5100C monitoring system (Medtronic, Minneapolis, MN, USA). Intraoperative oximetry data were analyzed beginning when participants entered the operating room in a non-blinded fashion. Given the relatively high incidence of neurologic complications (1, 8) and cerebrovascular impairment that may occur in cerebrovascular disease patients (18, 25), cerebral desaturation events may conceivably present a real risk to patients undergoing general anesthesia and surgery. Thus, we openly monitored intraoperative oximetry and utilized pre-established ventilatory strategies (26, 27) to mitigate desaturation events for safety purposes. The following safety protocol was implemented in the following stepwise manner:

- Ensure that mean arterial pressure was within 80% of preoperative baseline
- Increase the fraction of inspired oxygen to 100%, wait 5 min.
- Increase end-tidal carbon dioxide to 40–45 mmHg, wait 5 min.
- Consider packed red blood cell transfusion if appropriate as defined by institutional transfusion guidelines

Clinicians used their strategy of choice to increase blood pressure. Stepwise changes in ventilator parameters took place after 5 min, as respiratory-based changes in cerebral oximetry values generally take 5 min to equilibrate (28). We chose 3 min as an intervention time to assess for sustained events, rather than transient changes that may self-resolve. In fact, a sustained desaturation—based on a relative ≥20% decrease from baseline—has been shown to correlate with neurophysiologic evidence of ischemia after 4 min with 82.67% reliability, 100% sensitivity, and 82.83% specificity (15). Thus, the above safety interventions were implemented at 3 min, just prior to this 4-min threshold. No research outcomes were assessed in relation to these interventions—they were implemented for prophylactic patient safety purposes.

### Cognitive Function Testing

Cognitive function was assessed preoperatively and again once daily (mornings) during the first three postoperative days. The cognitive function testing battery was administered on an iPad platform (Joggle Research, Seattle, WA, USA) and took ~10–15 min to complete. The testing battery included the following six tests: Psychomotor Vigilance Test (Self-Test), Motor Praxis Test, Digit Symbol Substitution Test, Fractal-2-Back N-Back Test, Visual Object Learning Test, and Abstract Matching Test. Specific details regarding these tests, along with associated references, can be found in Supplementary Table 3. Scores for each individual test are automatically calculated based on speed and accuracy of response patterns, and individual test scores are then standardized and averaged to create composite scores per previously described methods (29, 30). Higher scores reflect better cognitive performance.

### Biomarker Analysis

Baseline blood samples were drawn in the immediate preoperative setting, and postoperative samples were drawn during scheduled morning phlebotomy rounds for the first three postoperative days. Blood specimens were collected into plastic tubes containing ethylenediaminetetraacetic acid for glial fibrillary acid protein processing, and the samples for S-100β, neuron specific enolase, and matrix metalloproteinase-9 were collected into plain plastic tubes containing no anticoagulant. Samples in plain tubes were allowed to clot at room temperature (at least 30 min) before processing, and samples were then centrifuged at 2000 g for 10 min at 4 degrees Celsius. Serum was separated into aliquots and either stored at −80 degrees Celsius (glial fibrillary acid protein, S-100β, matrix metalloproteinase-9) or refrigerated at 4 degrees Celsius (neuron specific enolase; max 7 days) prior to shipping. Specimens were shipped using frozen (glial fibrillary acid protein, S-100β, and matrix metalloproteinase-9) and refrigerated (neuron specific enolase) Category B biologic shipping systems per International Air Transport Association requirements.

Assay Performance was conducted at certified (Clinical Laboratory Improvement Amendment and College of American Pathologists) commercial and university laboratories. Serum S-100β levels were determined using a quantitative enzyme-linked immunosorbent assay (CanAg s100, ARUP Laboratories, Salt Lake City, UT, USA), with the lower limit of detection 12 ng/L. The glial fibrillary acid protein assay was performed using an electrochemiluminescent sandwich immunoassay as previously described at the Johns Hopkins University Pediatric Proteome Center (Baltimore, MD) (31). The lower limit of detection for this assay was 0.008 ng/mL, and the upper limit of detection was 40 ng/mL. For glial fibrillary acid protein values less than the lower limit of detection, the lower limit value was assigned; for values exceeding the upper limit of detection, the upper limit value was assigned. Neuron specific enolase serum concentrations were measured via homogenous automated immunofluorescent assay on the BRAHMS Kryptor (ThermoFisher Scientific, Waltham, MA, USA). The Kryptor uses Time Resolved Amplified Cryptate Emission based on non-radioactive transfer of energy. This assay was performed at Mayo Medical Laboratories, Rochester, MN, USA, and the lower limit of detection for the assay is 5 ng/mL. Lastly, matrix metalloproteinase-9 levels were determined via enzyme-linked immune assay (Colorado Coagulation, Esoterix, Englewood, CO, USA), with lower limit of detection 31 ng/mL.

### Statistical Analysis

Descriptive statistics were performed for all variables of interest. Continuous data were assessed for normality using the Shapiro-Wilks test. Parametric data were presented as mean (standard deviation) and non-parametric data were presented as median [25th percentile to 75th percentile]. Categorical data were presented as frequency (percentage). Univariate differences between the cerebrovascular disease group and control group were performed using independent *t*-tests or Mann-Whitney *U*-tests for continuous data and Chi-square or Fisher's Exact tests for categorical data, as appropriate. For comparing the incidence of cerebral desaturation events between groups, the relative risk (RR) was reported.

To assess the change in outcomes of interest over time, a series of linear repeated-measures generalized estimating equation models was constructed. Specifically, models were constructed for overall cognitive function test score and all four serum biomarker concentrations. This strategy allowed for analyzing longitudinal data over time (i.e., repeated measures) in the setting of partially missing data (32). Thus, all available data were included in models for both groups. Imputation was deferred, as cerebrovascular events could conceivably occur during the timeframe studied, which could significantly impact cognitive function and biomarker values. The models were constructed with main effects of time and group and an interaction term between time and group. If the interaction was non-significant, it was dropped from the model. An autoregressive correlation structure was used for all models. SAS version 9.4 (SAS Institute, Cary, NC USA) and IBM SPSS version 24 (Armonk, NY USA) were used for all analyses conducted. Statistical outliers were included in the analysis after validity confirmation. For neurocognitive function testing, this included post-test debriefs within the study team to ensure proper software functioning and test administration. For glial fibrillary acid protein values, samples were re-tested if there were any concerns, and laboratory meetings were held to review and confirm abnormally high samples.

For this study, the primary goal was to calculate a sample size that would allow detection of clinically relevant reductions in oximetry values as described previously. Given previous cerebral oximetry data in healthy patients (26) and in those with known cerebrovascular disease (33), a sample size of 18 per group provides >80% power to detect a relative 10% decrease from baseline values (alpha set to 0.05 with a two-sided paired *t*-test; mean of 63.9% with standard error of 9.2%). This conservative powering approach was adopted to provide an adequate sample size for detecting ≥20% decreases from baseline. Accounting for the possibility of missing data and participant withdrawal, 25 patients were recruited for each group. A *P* < 0.05, with two-tailed hypothesis testing, was considered statistically significant for all analyses.

## Results

After study initiation, 81 patients were screened and approached for study inclusion between May 2016 and September 2017. Within this group, 23 patients declined, one was found to be ineligible during screening, and one patient had baseline delirium, precluding consent. Of the 56 enrolled, five participants were withdrawn due to canceled surgeries, and one patient was found to not meet eligibility criteria shortly after enrollment. Baseline and demographic characteristics of both groups are presented in Table 1. Significantly more patients in the cerebrovascular group had coronary artery disease (13/25, 52%) compared to the control group (3/25, 12%) (*P* = 0.002). The nature and extent of cerebrovascular disease diagnosis for each participant is presented in the Supplemental Appendix (Supplementary Table 2).

**Table 1:** Baseline patient characteristics.

	**CVD (*n* = 25)**	**Control (*n* = 25)**	***P-*Value**
Age—years, mean (SD)	67 (10.9)	66 (12)	0.391
Male sex—no. (%)	12 (48)	12 (48)	1.000
Race—no. (%)			0.304
White	20 (80)	22 (88)	
Black	5 (20)	2 (8)	
Other	0 (0)	1 (4)	
**COMORBIDITIES—*****n*** **(%)**			
Stroke	17 (68)	–	
TIA	11 (44)	–	
CAS	6 (24)	–	
CAD	13 (52)	3 (12)	0.002
CHF	4 (16)	0 (0)	0.110
HTN	19 (76)	17 (68)	0.529
COPD	5 (20)	4 (16)	1.000
Atrial fibrillation	4 (16)	2 (8)	0.667
Aortic stenosis	3 (12)	0 (0)	0.235
CKD	8 (32)	4 (16)	0.185
DM	10 (40)	6 (24)	0.225
Type of surgery—*n* (%)			1.000
Open GI	9 (36)	9 (36)	
Open GU	4 (16)	4 (16)	
Cervical fusion	4 (16)	4 (16)	
TAH-BSO	3 (12)	3 (12)	
Thoracolumbar fusion	2 (8)	2 (8)	
Hepatobiliary	2 (8)	2 (8)	
Shoulder arthroplasty	1 (4)	1 (4)	

*SD, standard deviation; no., number; CVD, cerebrovascular disease; TIA, transient ischemic attack; CAS, carotid artery stenosis; CAD, coronary artery disease; CHF, congestive heart failure; HTN, hypertension; COPD, chronic obstructive pulmonary disease; CKD, chronic kidney disease; DM, type 2 diabetes mellitus; GI, gastrointestinal; GU, genitourinary; TAH-BSO, total abdominal hysterectomy with bilateral salpingo-oophorectomy*.

### Cerebral Oximetry and Intraoperative Physiology

Intraoperative data were removed from one participant in each group—poor signal quality was noted in one of the cerebrovascular disease patients, and the surgeon requested oximeter sensor removal in the corresponding control patient. This brought the total number of participants to 24 for intraoperative oximetry data analysis (Table 2). Both baseline and minimum cerebral oximetry values were similar between the groups. Sustained desaturation events occurred 7/24 (29%) patients in the cerebrovascular group compared to 2/24 (8.3%) in the control group (RR 3.5, 95% CI 0.81–15.2; *P* = 0.094). Given the effects of intraoperative physiology on cerebral oximetry values (26), physiologic profiles were also compared between the groups during surgery (inspired oxygen was, at times, slightly higher in the cerebrovascular disease group, though this difference [−2.0% fraction of inspiratory oxygen] was unlikely to be of clinical significance). No significant physiologic differences were otherwise observed (Supplementary Table 4).

**Table 2:** Cerebral oximetry comparisons between groups.

**Cerebral oximetry data**	**CVD group**	**Control group**	***P-*value**
	**(*N* = 25)**	**(*N* = 25)**	
**BASELINE rSO**_**2**_**–MEAN% (SD)**			
Left	62 (8.8)	64 (6.7)	0.431
Right	62 (8.7)	63 (7.9)	0.427
Minimum rSO_2_–mean% (SD)	53 (7.2)	55 (7.1)	0.190
Desaturation event^*^–n (%)	7 (29)	2 (8.3)	0.094

* *Desaturation event was defined as a sustained decrease below the specified thresholds (≥20% from baseline or < 50% absolute) for at least 3 min. A relative risk ratio, compared to the control group, was calculated for desaturation events. Intraoperative data were only included for 24 patients in each group. CVD, cerebrovascular disease; rSO_2_, regional cerebral oximetry; SD, standard deviation*.

### Neurocognitive Evaluation

**Figure 1:**
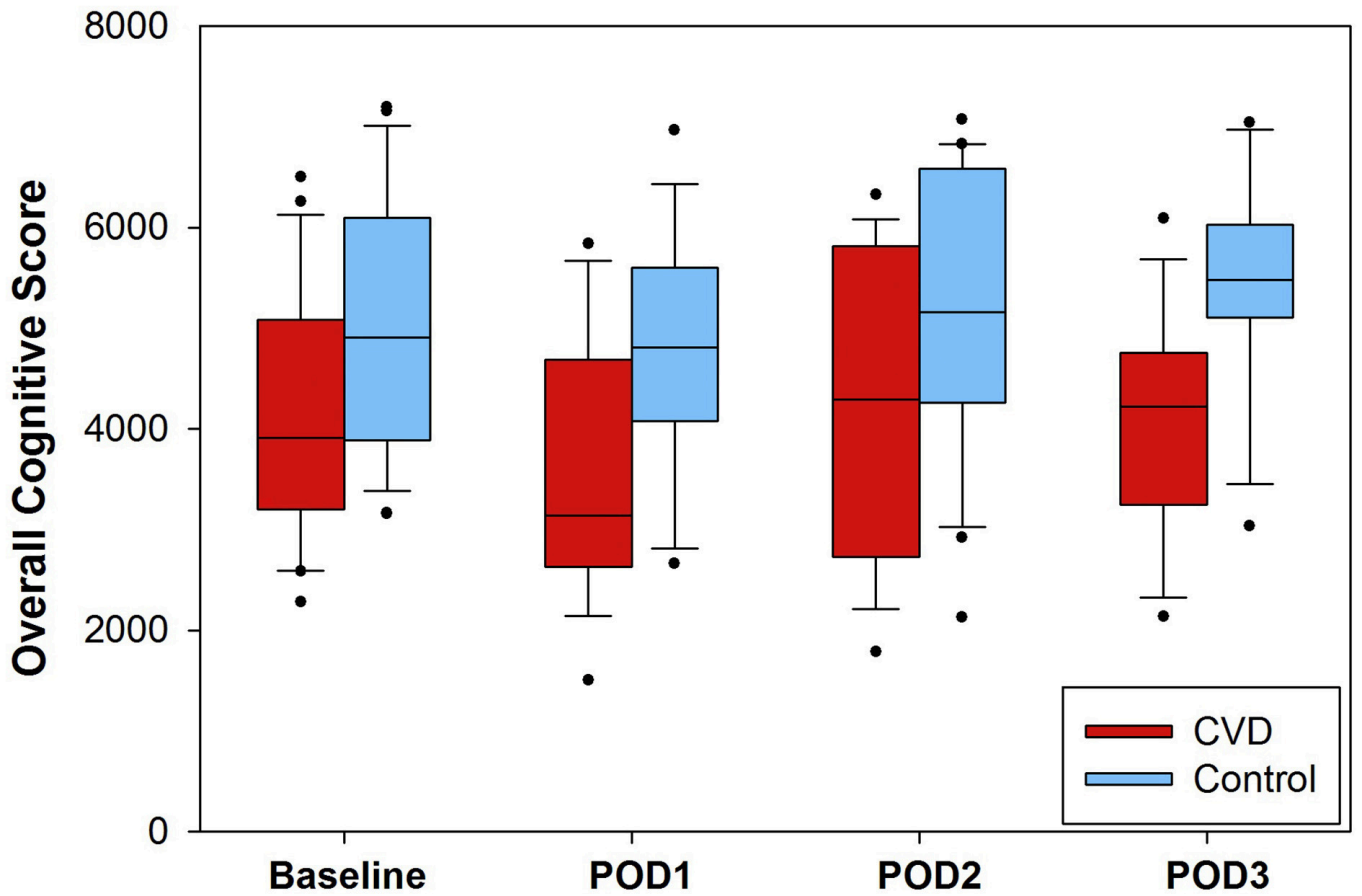
Composite cognitive function scores presented for each group. Higher scores were reflective of higher cognitive function. The interaction of group and time was not significant per generalized estimated equation modeling and was thus removed from the model. After adjusting for time, the cerebrovascular disease (CVD) group demonstrated significantly lower overall scores (approximately one standard deviation) compared to the control group (−1049, 95% CI: −1662, −436; *P* < 0.001). The number of daily assessments available were as follows: baseline, *N* = 24 (CVD group) and *N* = 24 (control group); POD1, *N* = 18 (CVD group) and *N* = 19 (control group); POD2, *N* = 19 (CVD group), and *N* = 20 (control group); POD3, *N* = 13 (CVD group) and *N* = 14 (control group). POD = postoperative day.

At the beginning of each neurocognitive evaluation, modified National Institutes of Health Stroke Scale scores were calculated. In the cerebrovascular disease group, 8/25 (32%) had increases in scores from baseline, and 5/25 (20%) matched controls also had increases from baseline (Supplementary Figure 1; group-level comparison, *P* = 0.328). After completion of stroke scale evaluation, the cognitive function testing battery was performed. Daily scores (range: 1500–7197 points) are presented in Figure 1 for each group. There was no significant interaction between group and time, so the interaction term was removed from the model. After adjusting for time, composite scores were ~1 standard deviation lower in the cerebrovascular disease group throughout the entire perioperative period (−1049 [95% CI −1661, −436], *P* < 0.001). *Post-hoc*, exploratory analysis revealed no significant difference in cognitive function scores between the group experiencing an intraoperative desaturation event and those not experiencing intraoperative desaturation (Supplementary Table 5).

### Serum Biomarkers

**Figure 2:**
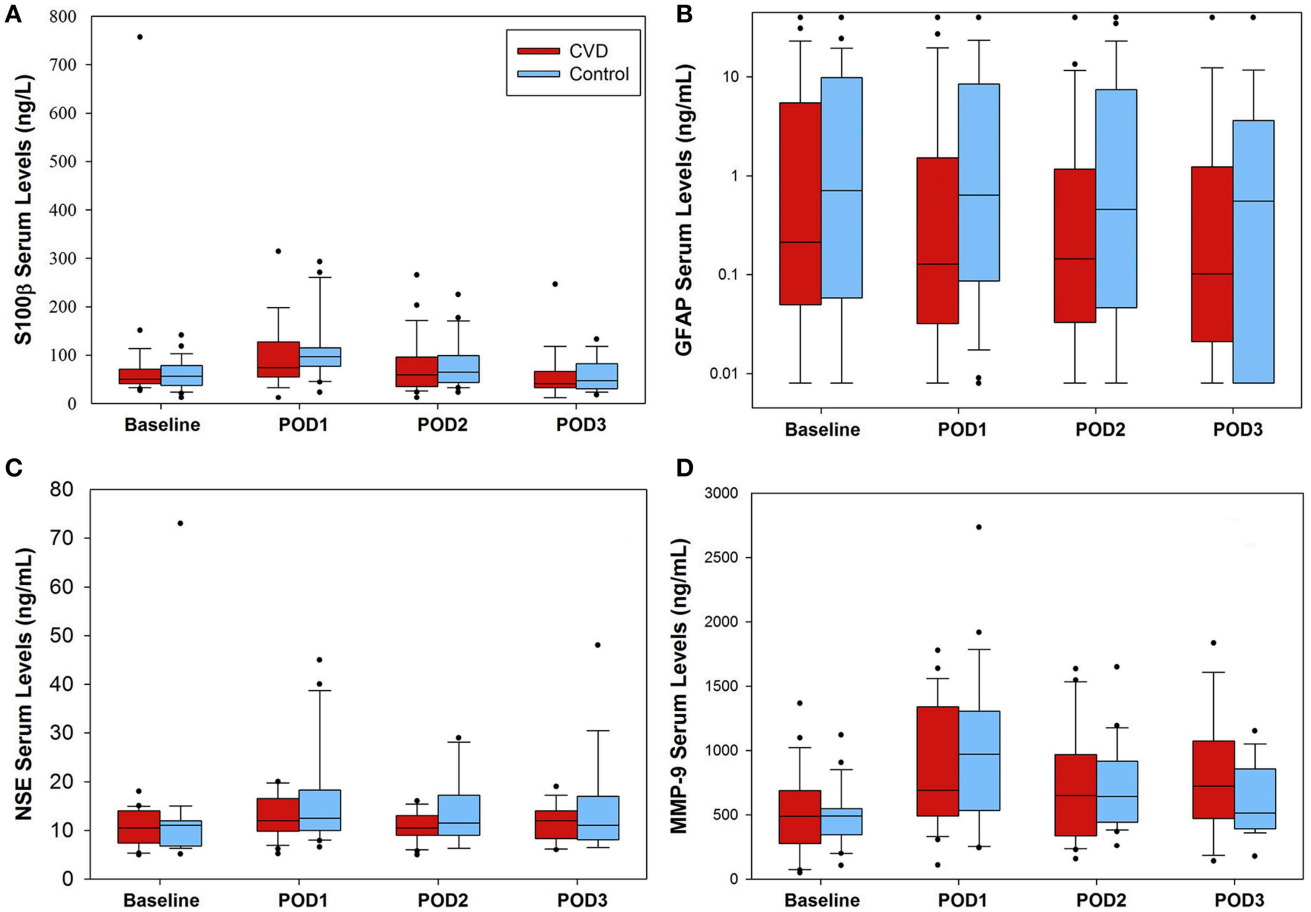
Serum biomarker concentrations in the perioperative setting. No significant differences between groups were found for any of the biomarkers tested. **(A)** S-100β data presented. For the cerebrovascular disease (CVD) group, number of samples were as follows (baseline through POD3): 25, 25, 24, 19, respectively; control group: 25, 25, 24, 19. **(B)** glial fibrillary acid protein (GFAP) levels varied considerably within both groups, ranging from 0.008 to 40 ng/mL. Number of CVD group daily samples: 25, 24, 24, 19; control group samples: 25, 25, 24, 18. **(C)** Daily neuron specific enolase (NSE) serum levels. Number of CVD group daily samples: 20, 22, 22, 15; control group: 19, 20, 18, 16. Lastly, **(D)** matrix metalloproteinase-9 (MMP-9) data presented. Number of CVD group daily samples: 25, 25, 24, 17. Control group daily samples: 23, 24, 22, 17. POD = postoperative day.

Daily serum biomarker results are presented in Figure 2 for both groups. There were no significant time interaction terms for any of the biomarkers; thus, interaction terms were removed from all models. After accounting for time, compared to the control group, there were no significant differences in S-100β (ng/L; 5.5 [95% CI −25.4, 36.5], *P* = 0.726), glial fibrillary acid protein (ng/mL; −1.29 [95% CI −6.3, 3.7], *P* = 0.615), neuron specific enolase (ng/mL; −3.0 [95% CI −7.2, 1.3], *P* = 0.169), or matrix metalloproteinase-9 (ng/mL; 30.9 [95% CI −138, 199], *P* = 0.719) in the cerebrovascular disease group. *Post-hoc*, exploratory analysis revealed no significant differences in any of the serum biomarkers studied between intraoperative desaturation and non-desaturation groups (Supplementary Table 5).

### Documented Hypoxic-Ischemic Injury

**Figure 3:**
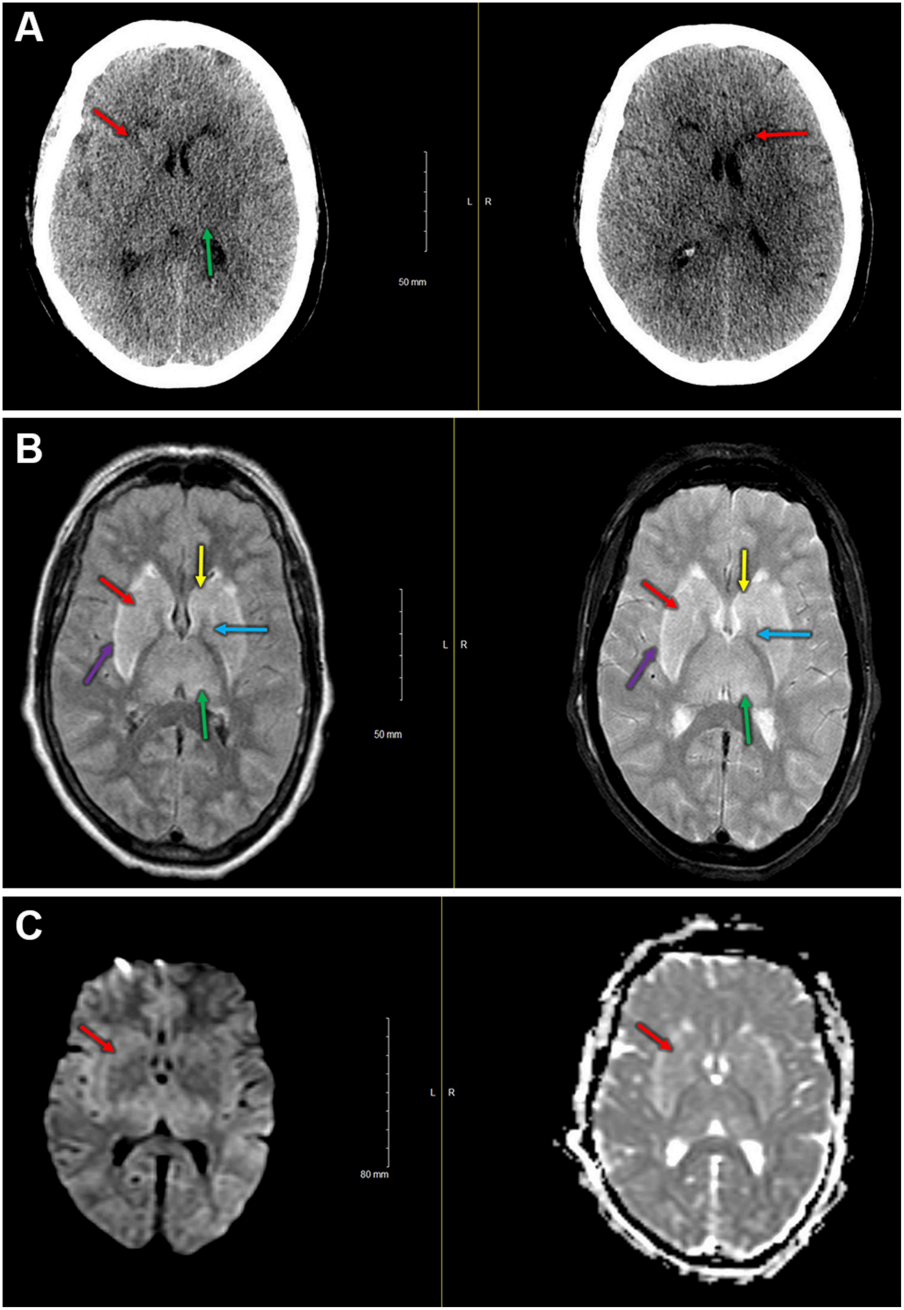
**(A)** Immediate postoperative non-contrast head CT shows bilateral symmetric hypoattenuation adjacent to the basal ganglia (red arrows). Less pronounced findings throughout the thalamus (green arrow). **(B)** Left—fluid attenuation inversion recovery imaging shows bilateral hyperintense signals in the basal ganglia (caudate [yellow] and putamen [red], globus pallidus [blue]) and in the thalami [green]). Note the hyperintense signal at the external capsule [purple], just lateral to the putamina. Right—T2 fat suppressed imaging demonstrating hyperintense signal in the basal ganglia (caudate [yellow] and putamen [red], globus pallidus [blue]) and in the thalami [green]). Hyperintense signal also present in the external capsules [purple]. **(C)** Diffusion-weighted imaging (left) and apparent diffusion coefficient (right) demonstrate slightly elevated signal on diffusion-weighted imaging with small areas of hypointense signal on the apparent diffusion coefficient in the basal ganglia (i.e., right anterior putamen, red arrow).

One participant in the control group experienced diffuse, bilateral cerebral injury intraoperatively with neuroradiological evidence most suggestive of hypoxic-ischemic etiology. This participant was a 76-year-old female with a history of atrial fibrillation (on rivaroxaban, held for 3 days preoperatively) and hypertension who presented for a two-level thoracic decompression and fusion. The case proceeded uneventfully, and after the surgical dressing was complete, no purposeful movement was witnessed for ~40 min. After this time, the patient appeared to develop generalized, rhythmic tonic-clonic movements concerning for seizure. This activity was terminated with propofol, and the patient was taken for an urgent non-contrast head CT scan. The scan demonstrated bilateral symmetric hypoattenuation adjacent to the basal ganglia (Figure 3A). The ensuing brain MRI, obtained ~12 h later, revealed symmetric elevated fluid-attenuation inversion recovery and T2 signal in the basal ganglia, external capsules, and thalami (Figure 3B). Areas of elevated diffusion-weighted imaging signal (with corresponding hypointense signal on apparent diffusion coefficient) are also noted (Figure 3C). The patient remained deeply obtunded for several days, and after gradual, modest improvement, she was ultimately discharged to a long-term acute care facility 2 weeks later. In terms of study measures, the baseline oximetry values were 67% (left) and 68% (right), and the patient experienced a non-sustained desaturation event (< 1 min) with minimum oximetry value 54% ~1 h prior to the seizure. Serum biomarker data for this patient are presented in Table 3.

**Table 3:** Serum biomarkers for the participant (*n* = 1) with documented hypoxic-ischemic injury.

**Biomarkers**	**Baseline**	**POD1**	**POD2**	**POD3**
S-100β (ng/L)	59	97	110	49
GFAP (ng/mL)	0.536	0.271	0.369	0.367
NSE (ng/mL)	6.8	7.9	6.6	6.5
MMP-9 (ng/mL)	322	259	723	379

*POD, postoperative day; GFAP, glial fibrillary acid protein; NSE, neuron specific enolase; MMP-9, matrix metalloproteinase-9*.

## Discussion

The objective of this study was to characterize perioperative neurologic vulnerability, particularly for stroke, using surrogate endpoints related to the cerebrovascular system: cerebral oximetry, cognitive function, and molecular biomarkers. These outcomes were studied in relatively high-risk, cerebrovascular disease patients and matched controls. We found that pre-defined cerebral desaturation events occurred in both the cerebrovascular disease and control groups. Those with cerebrovascular disease demonstrated significantly worse cognitive function throughout the entire perioperative period; however, postoperative trajectories were similar compared to the control group. No significant differences were found among serum biomarkers between the groups. Lastly, neither the pre-defined cerebral oximetry thresholds nor the chosen serum biomarkers correlated with confirmed hypoxic-ischemic injury sustained by a single patient in the control group.

With a limited sample size—and the relatively low incidence of perioperative stroke and related events—this study might otherwise preclude definitive conclusions. However, one participant in the control group did experience significant bilateral hypoxic-ischemic injury perioperatively, and neither the pre-specified oximetry thresholds nor biomarker strategies were able to predict or detect this injury. Oximetry values did decrease to 20% below baseline but for <1 min. With this participant, the pre-defined oximetry strategy may not have been sensitive enough to detect the injury. In fact, recent evidence demonstrates that the magnitude of time spent below a 10% relative decrease correlates with MRI findings of ischemia after cardiac surgery (34). Additionally, the nature of the injury might not have been detected by the near-infrared spectroscopy methodology. Indeed, pathological neuroimaging findings were noted in subcortical regions, and commercially available cerebral near-infrared spectroscopy devices penetrate tissue to ~1.5–2 cm (35). Serum biomarker changes were also not dramatically increased (Table 3). It is surprising that serum biomarkers did not increase above general postoperative averages given the severity of injury that occurred. Ultimately, these currently tested methods—pre-defined absolute cerebral oximetry thresholds, serum biomarkers (S-100β, glial fibrillary acid protein, and matrix metalloproteinase-9), and sophisticated neurocognitive testing, were unable to predict, detect, or prevent severe brain injury postoperatively.

Additional inferences can be made by reflecting on the observational findings across both cohorts. Surgical patients with pre-existing cerebrovascular disease frequently experienced pre-defined cerebral desaturation events with an incidence approaching 30%. However, the clinical relevance of these findings remains in question. As discussed, the 3-min, pre-specified thresholds were inadequate for detecting the major hypoxic-ischemic injury that did occur. Nonetheless, cerebrovascular disease patients may still demonstrate lower perioperative cerebral oximetry trends: there was a 3.5-fold increase in cerebral desaturation incidence, but this difference was not statistically significant (*P* = 0.094) when compared to the control group. This may have been due to Type II error given that this study was not powered for direct group comparisons. Indeed, cerebrovascular disease is associated with impaired autoregulation (17) and misery perfusion (36), and monitoring cerebral flood flow may require more dynamic strategies, rather than relying on static, imprecise physiologic thresholds. For example, real-time correlation analysis with cerebral oximetry and mean arterial blood pressure may allow for individual autoregulation monitoring intraoperatively (37). Surgical patients with pre-existing cerebrovascular disease may benefit from such dynamic cerebrovascular monitoring strategies, given that absolute oximetry thresholds are difficult to establish (34). Cognitive function scores were also lower in the cerebrovascular disease group across the entire perioperative period. Trends were similar between groups (i.e., no significant postoperative changes from baseline), though the lower cognitive reserve in cerebrovascular disease patients supports the notion that cerebrovascular disease may predispose to postoperative neurocognitive disorders (38). Lastly, there were no significant differences in serum biomarker levels between groups, and perioperative variability was high among all proteins tested. Surprisingly, glial fibrillary acid protein values also varied widely across participants (<0.008 ng/mL to >40 ng/mL). Non-neurologic causes may have driven high values in certain participants. Glial fibrillary acid protein expression has been associated with inflammatory bowel disease (39–41) and various forms of carcinoma (42, 43), and multiple patients in this study with high perioperative values (>9 ng/mL) had histories of inflammatory bowel disease and/or various uro-gynecologic malignancies. These findings call into question the sensitivity and specificity of glial fibrillary acid protein for detecting brain injury (22). Further foundational work is needed to better identify biomarkers that are (1) highly specific to brain cells (e.g., neurons, glia), (2) detectable in the serum, and (3) elevated in direct response to brain/cerebrovascular injury. It does not appear that these criteria were met with our patient who experienced significant hypoxic-ischemic injury, as previously described.

### Strengths and Limitations

Strengths of the study design include the multidimensional neurologic assessment, consistency of anesthetic technique, and use of a matched control group. There are also notable limitations with this study. The study was powered to detect desaturation events within the cerebrovascular disease group, and the resulting sample size was small. As such, the conclusions that can be drawn from these findings are limited given the small sample size. However, as discussed, one control patient did experience significant bilateral, diffuse hypoxic-ischemic injury without significant biomarker changes. Thus, the proposed biomarkers in this study may have limited utility for detecting such events. These insights can, however, be used to inform future candidate biomarker studies for perioperative hypoxic-ischemic injury. Cerebrovascular disease inclusion criteria were intentionally broad, particularly for carotid stenosis, for reasons previously described. Indeed, neither the degree of carotid stenosis, nor the presence of related symptoms, were considered in this study. Future studies can focus on particular subgroups as appropriate. Our safety intervention strategy for treating cerebral desaturation events may also have blunted downstream consequences that could have otherwise occurred, limiting our positive findings. Additionally, clinicians and research team members in the OR were not blinded to the oximetry data collection, for reasons mentioned in the Materials and Methods section. Missing cognitive function and biomarker data were present, though the proportion of daily missing values was similar between groups (see legends, Figures 1, 2). Lastly, delirium was not specifically addressed in this study.

In conclusion, while there may be a trend toward increased cerebral desaturation events in the cerebrovascular disease group, there were otherwise no dramatic differences in cognitive function trends or plasma biomarkers compared to controls. A catastrophic cerebrovascular event was not associated with sustained cerebral desaturation or any biomarker signature of injury. These data highlight the challenges with assessing patients at risk for cerebrovascular events and emphasize the need for further study to better understand and prevent perioperative stroke and related injury.

## Ethics Statement

This study was carried out in accordance with the University of Michigan Medical School Institutional Review Board (HUM00106530, approved 11/2/2016). All subjects gave written informed consent in accordance with the Declaration of Helsinki. The protocol was approved by the University of Michigan Medical School Institutional Review Board.

## Author Contributions

PV, BK, AT, MS, AM, GM, and PP: study conception and design. PV, BK, MZ, MS, MC, and AM: participant recruitment. PV, BK, MZ, RL, MS, MC, and AM: data acquisition. PV, BK, AT, RL, AE, GM, and PP: data analysis. PV and AT: statistical support. All authors: manuscript preparation, have reviewed the manuscript for intellectual content, approved the final version, and agree to be accountable for all aspects of the work.

### Conflict of Interest Statement

AE serves as a consultant for ImmunArray, LTD. The remaining authors declare that the research was conducted in the absence of any commercial or financial relationships that could be construed as a potential conflict of interest.
