# Supplementary material for: Cerebrovascular Disease and Perioperative Neurologic Vulnerability: A Prospective Cohort Study
Source: Front Neurol. 2019 May 28;10:560. doi: 10.3389/fneur.2019.00560 (PMC6558425; doi:10.3389/fneur.2019.00560)
Supplement: Supplementary file 2 [file Table_2.DOCX]

**Supplementary Table 2.** Cerebrovascular Disease History

| **Participant** | **Age (years)** | **Sex** | **Race** | **CVD History** | **mRS** | **Preop mNIHSS** | **Base**  **rSO_2_- L** | **Base rSO_2_-**  **R** | **Time since stroke (months)** | **Stroke Territory** | **TOAST Classification** | **Source Documentation** |
| --- | --- | --- | --- | --- | --- | --- | --- | --- | --- | --- | --- | --- |
| 1 | 75 | F | White | Stroke | 2 | 2 | 51 | 56 | >12 to 24 | Left MCA, L occipital, L thalamic, and L cerebellar infarcts | Cryptogenic | Outside records |
| 2 | 73 | M | White | Bilateral CAS | -- | 0 | 67 | 64 | -- | -- | -- | Outside records |
| 3 | 43 | F | African-American | Stroke | 1 | 0 | 55 | 51 | >24 | L MCA | Cryptogenic | Outside records |
| 4 | 74 | M | White | Stroke, TIA | 0 | 0 | 55 | 64 | >24 | R IC, L thalamic | Not reported | UMHS records |
| 5 | 73 | F | White | Bilateral CAS | -- | 0 | 44 | 41 | -- | -- | -- | Outside records |
| 6 | 69 | M | African-American | TIA | -- | 0 | 68 | 68 | -- | -- | -- | Outside records |
| 7 | 67 | F | White | Stroke | 1 | 2 | 53 | 53 | >24 | R MCA, R subcortical | Small-vessel occlusion | Outside records |
| 8 | 63 | F | African-American | Stroke | 2 | 0 | 73 | 74 | >24 | L MCA | Undetermined | Outside records |
| 9 | 53 | F | White | Stroke | 2 | 4 | 69 | 65 | >24 | Unknown* | Other (Anti-cardiolipin Ab) | Patient history |
| 10 | 63 | M | African-American | Stroke | 2 | 4 | 55 | 47 | >24 | L PCA | Not reported | UMHS records |
| 11 | 72 | F | White | TIA, CAS | -- | 1 | 52 | 58 | -- | -- | -- | Outside records |
| 12 | 69 | M | White | Stroke | 2 | 0 | 73 | 74 | Unknown^†^ | L basal ganglia, L corona radiata | Small-vessel occlusion | UMHS records |
| 13 | 69 | M | White | TIA | -- | 1 | 64 | 76 | -- | -- | -- | UMHS records, outside records |
| 14 | 74 | F | White | Stroke | 4 | 0 | 56 | 62 | >24 | Not reported | Not reported | Outside records, patient history |
| 15 | 65 | M | White | Stroke, TIA | 0 | 0 | 63 | 62 | >24 | Unknown* | Unknown* | Outside records, patient history |
| 16 | 58 | F | White | Stroke | 0 | 0 | 64 | 59 | Unknown* | Unknown* | Unknown* | Outside records |
| 17 | 71 | M | White | Severe L CAS | -- | 0 | 60 | 60 | -- | -- | -- | Outside records |
| 18 | 51 | M | White | Stroke | 0 | 3 | 59 | 64 | <9 | R MCA | Cardioembolism | UMHS records |
| 19 | 85 | F | White | Stroke | 5 | 16 | 53 | 54 | >24 | L MCA | Not reported | UMHS records |
| 20 | 78 | M | African-American | Stroke | 0 | 0 | 56 | 53 | >24 | Unknown* | Unknown* | Patient history |
| 21 | 80 | F | White | Stroke, TIA | 2 | N/A | 72 | 70 | >24 | R external capsular | Small-vessel occlusion | Outside records |
| 22 | 57 | M | White | TIA | -- | 0 | 71 | 72 | -- | -- | -- | UMHS records, patient history |
| 23 | 69 | F | White | Stroke, TIA | 0 | 0 | 79 | 64 | >24 | Not reported | Not reported | UMHS records, patient history |
| 24 | 42 | F | White | Stroke | 0 | 0 | 63 | 67 | >24 | Multiple bilateral cortical lesions | Not reported | Outside records |
| 25 | 75 | M | White | TIA | -- | 0 | 72 | 61 | -- | -- | -- | UMHS records, patient history |

CVD = cerebrovascular disease, mRS = Modified Rankin Scale, preop = preoperative, mNIHSS = modified National Institutes of Health Stroke Scale, Base = baseline, rSO_2_ = regional cerebral oximetry (L = left, R = right), TOAST = Trial of Org 10172 in Acute Stroke Treatment stroke classification system, MCA = middle cerebral artery, CAS = carotid artery stenosis, TIA = transient ischemic attack, IC = internal capsule, PCA = posterior cerebral artery, UMHS = University of Michigan Health System, Ab = antibody. ^†^Age indeterminate on neuroimaging, *unable to obtain original medical records.
