# Supplementary material for: Cerebrovascular Disease and Perioperative Neurologic Vulnerability: A Prospective Cohort Study
Source: Front Neurol. 2019 May 28;10:560. doi: 10.3389/fneur.2019.00560 (PMC6558425; doi:10.3389/fneur.2019.00560)
Supplement: Supplementary file 3 [file Table_3.DOCX]

**Supplementary Table 3.** Cognitive Function Tests

| **Test** | **Description** | **References** |
| --- | --- | --- |
| Psychomotor Vigilance Test – Self-Test | Measures sustained or vigilant attention by recording reaction times to visual stimuli that occur at random inter-stimulus intervals. Able to track changes in behavioural alertness without the confounding effects of aptitude and learning. It is validated to be sensitive to acute total sleep loss, chronic sleep restriction, and a variety of other fatigue-inducing factors. The duration of the test in this study was 3 minutes, which has similar sensitivity and specificity compared to the 10-minute version | Basner et al., *Sleep* 2011; 34: 581-91 (1) |
| Motor Praxis Test | This test measures sensory-motor speed. During the test, participants touch a shrinking green box on the iPad screen and continue to touch the box each time it appeared on a different location on the test-screen | Gur et al., *J Neurosci Methods* 2010; 187: 254-62 (2) |
| Digit Symbol Substitution Test | This involves matching numbers to symbols and is a measure of complex scanning, visual tracking, and attention. The iPad screen shows a key with a set of ten symbols and their corresponding digits (0 through 9). When a symbol is presented on the screen, participants are required to touch its corresponding number on the test-screen. This task has gained widespread use in the investigation and modelling of sleep-wake regulatory processes. | Joy et al., *Assessment* 2003; 10: 56-65 (3) |
| Fractal-2-Back (N-Back) | Measures attention and working memory. Participants are asked to pay attention to fractal images on the iPad screen, one at a time, and to touch the screen whenever the image on the screen is the same as the image before the previous one. | Ragland et al., *Neuropsychology* 2002; 16: 370-9 (4) |
| Visual Object Learning Test | This test examines aspects of visual-spatial learning and memory in a manner analogous to available verbal tests. Participants learn a set of visual stimuli by viewing the set of stimuli on the iPad screen repeatedly, and then be asked to determine if a given object was in the set of stimuli presented | Glahn et al., *Neuropsychology* 1997; 11: 602-12 (5) |
| Abstract Matching Test | The abstract matching test is a series-completion test that examines abstraction and cognitive flexibility. Participants are presented with a pair of shapes, potentially varying in colour and in shape, as well as two options of shape pairs. Participants must determine the implicit abstract rule to choose the correct shape pair that completes the series | Albert et al., *Psychol Aging* 1990; 5: 94-100 (6) |

Individual test scores are automatically calculated based on speed and accuracy of response. These test scores are then standardised and averaged to create composite scores as described in the manuscript. Higher scores are reflective of higher cognitive function (Score range [points]: 1500 – 7197, mean: 4554, standard deviation: 1373). The number of daily assessments available were as follows: baseline, N = 24 (CVD group) and N = 24 (control group); POD1, N = 18 (CVD group) and N = 19 (control group); POD2, N = 19 (CVD group) and N = 20 (control group); POD3, N = 13 (CVD group) and N = 14 (control group). POD = postoperative day

**Supplemental References**

1. Basner, M, and Dinges, DF. Maximizing sensitivity of the psychomotor vigilance test (PVT) to sleep loss. *Sleep* 34 (2011) 581-91.

2. Gur, RC, Richard, J, Hughett, P, Calkins, ME, Macy, L, Bilker, WB, et al. A cognitive neuroscience-based computerized battery for efficient measurement of individual differences: Standardization and initial construct validation. *J Neurosci Methods* 187 (2010) 254-62.

3. Joy, S, Fein, D, and Kaplan, E. Decoding digit symbol: Speed, memory, and visual scanning. *Assessment* 10 (2003) 56-65.

4. Ragland, JD, Turetsky, BI, Gur, RC, Gunning-Dixon, F, Turner, T, Schroeder, L, et al. Working memory for complex figures: An fMRI comparison of letter and fractal n-back tasks. *Neuropsychology* 16 (2002) 370-9.

5. Glahn, DC, Gur, RC, Ragland, JD, Censits, DM, and Gur, RE. Reliability, performance characteristics, construct validity, and an initial clinical application of a visual object learning test (volt). *Neuropsychology* 11 (1997) 602-12.

6. Albert, MS, Wolfe, J, and Lafleche, G. Differences in abstraction ability with age. *Psychol Aging* 5 (1990) 94-100.
