# Supplementary material for: Cerebrovascular Disease and Perioperative Neurologic Vulnerability: A Prospective Cohort Study
Source: Front Neurol. 2019 May 28;10:560. doi: 10.3389/fneur.2019.00560 (PMC6558425; doi:10.3389/fneur.2019.00560)
Supplement: Supplementary file 4 [file Table_4.DOCX]

**Supplementary Table 4**. Intraoperative Physiological Measures by Quarter of Case Duration

|  | **First Quarter of Case Duration** | | | **Second Quarter of Case Duration** | | |
| --- | --- | --- | --- | --- | --- | --- |
|  | **Control** | **CVD** | **P-Value** | **Control** | **CVD** | **P-Value** |
| **Physiologic Measures** | | | | | | |
| Systolic Pressure | 106.0 [102.0 to 122.0] | 116.0 [106.0 to 137.0] | 0.070 | 118.2 (16.2) | 122.6 (13.7) | 0.305 |
| Diastolic Pressure | 61.4 (9.1) | 61.3 (11.2) | 0.962 | 64.0 (9.1) | 61.4 (10.5) | 0.369 |
| Mean Arterial Pressure | 80.1 (11.4) | 83.0 (13.1) | 0.410 | 84.4 (10.0) | 83.2 (9.8) | 0.670 |
| Heart Rate | 73.5 (10.4) | 70.6 (13.7) | 0.391 | 73.5 (11.8) | 69.0 (12.3) | 0.189 |
| End-Tidal CO2 | 33.5 (2.2) | 33.7 (2.2) | 0.766 | 34.2 (2.1) | 33.9 (2.4) | 0.611 |
| Measured FiO2 | 50.0 [50.0 to 50.0] | 50.0 [50.0 to 50.0] | 0.812 | 50.0 [50.0 to 50.0] | 50.0 [50.0 to 55.0] | 0.070 |
| SpO2 Percentage | 98.0 [96.5 to 99.0] | 98.0 [97.0 to 99.0] | 0.385 | 97.5 [96.0 to 99.0] | 98.0 [97.0 to 99.0] | 0.562 |
| Inspired Oxygen | 44.5 [43.0 to 46.0] | 45.5 [44.0 to 50.0] | 0.145 | 43.0 [42.0 to 45.0] | 45.0 [44.0 to 47.0] | 0.031 |
| Temperature | 36.0 (0.4) | 35.9 (0.5) | 0.784 | 36.1 (0.5) | 36.0 (0.5) | 0.682 |
| **Gasses Used** | | | | | | |
| Isoflurane | 0.8 [0.5 to 0.9] | 0.8 [0.7 to 1.0] | 0.179 | 0.8 [0.6 to 1.0] | 0.9 [0.8 to 1.0] | 0.119 |
| Sevoflurane | 1.8 (0.0) | 1.0 (0.8) | 0.582 | 1.5 (0.0) | 1.6 (0.0) | NA |
| Nitrous Oxide | 0.0 [0.0 to 0.0] | 0.0 [0.0 to 0.0] | 0.643 | 0.0 [0.0 to 0.0] | 0.0 [0.0 to 0.0] | 0.607 |
| Age-adjusted MAC | 0.9 (0.2) | 0.9 (0.1) | 0.518 | 1.0 (0.2) | 1.0 (0.1) | 0.908 |
|  | | | | | | |
|  | **Third Quarter of Case Duration** | | | **Fourth Quarter of Case Duration** | | |
|  | **Control** | **CVD** | **P-Value** | **Control** | **CVD** | **P-Value** |
| **Physiologic Measures** | | | | | | |
| Systolic Pressure | 113.5 [103.0 to 128.0] | 115.0 [108.0 to 131.0] | 0.413 | 117.4 (18.7) | 125.5 (14.1) | 0.091 |
| Diastolic Pressure | 62.8 (8.2) | 61.6 (8.4) | 0.614 | 62.9 (9.4) | 63.2 (9.7) | 0.898 |
| Mean Arterial Pressure | 83.0 (10.5) | 82.2 (8.0) | 0.755 | 83.4 (12.2) | 85.2 (9.3) | 0.571 |
| Heart Rate | 70.5 [61.0 to 83.0] | 68.5 [61.0 to 80.0] | 0.576 | 73.9 (11.4) | 73.2 (11.8) | 0.678 |
| End-Tidal CO2 | 34.1 (2.7) | 34.2 (2.2) | 0.953 | 35.6 (3.2) | 35.5 (2.9) | 0.851 |
| Measured FiO2 | 50.0 [50.0 to 50.0] | 50.0 [50.0 to 50.0] | 0.740 | 50.0 [50.0 to 50.0] | 50.0 [50.0 to 50.0] | 0.545 |
| SpO2 Percentage | 99.0 [97.0 to 99.0] | 98.0 [97.0 to 99.0] | 0.616 | 98.0 [97.0 to 99.0] | 98.0 [97.0 to 99.0] | 0.716 |
| Inspired Oxygen | 43.0 [42.0 to 45.0] | 45.0 [43.0 to 46.0] | 0.042 | 44.5 [42.0 to 47.0] | 45.0 [43.0 to 47.0] | 0.390 |
| Temperature | 36.3 [36.1 to 36.9] | 36.4 [35.7 to 36.8] | 0.456 | 36.7 [36.3 to 37.0] | 36.5 (0.6) | 0.586 |
| **Gasses Used** | | | | | | |
| Isoflurane | 0.8 (0.2) | 0.9 (0.3) | 0.176 | 0.7 [0.4 to 0.9] | 0.7 [0.5 to 0.9] | 0.643 |
| Sevoflurane | 1.5 (0.0) | 1.5 (0.0) | NA | 1.1 (0.0) | 1.3 (0.0) | NA |
| Nitrous Oxide | 0.0 [0.0 to 0.0] | 0.0 [0.0 to 0.0] | 0.574 | 0.0 [0.0 to 0.0] | 0.0 [0.0 to 0.0] | 0.973 |
| Age-adjusted MAC | 1.0 [0.9 to 1.0] | 1.0 [0.9 to 1.0] | 0.946 | 0.8 [0.7 to 0.9] | 0.8 [0.7 to 0.9] | 0.854 |

Data are presented as mean (standard deviation) with an independent t-test p-value or median [25^th^ percentile to 75^th^ percentile] with a Mann-Whitney U test p-value, as appropriate. CO2 = carbon dioxide; MAC = minimum alveolar concentration, FiO2 = fraction of inspired oxygen, SpO2 = blood oxygen saturation, CVD = cerebrovascular disease. Case duration was measured from Anaesthesia Induction End to Surgical Incision.
