# Supplementary material for: Cerebrovascular Disease and Perioperative Neurologic Vulnerability: A Prospective Cohort Study
Source: Front Neurol. 2019 May 28;10:560. doi: 10.3389/fneur.2019.00560 (PMC6558425; doi:10.3389/fneur.2019.00560)
Supplement: Supplementary file 5 [file Table_5.DOCX]

**Supplementary Table 5.** Comparisons Between Desaturation (n=9) and Non-Desaturation Groups (n=39)

| **Outcome** | **β Estimate** | **95% Confidence Interval** | ***P-value*** |
| --- | --- | --- | --- |
| Cognitive Function Score (points, n): | -645 | -1659, 369 | 0.213 |
|  |  |  |  |
| S-100β (ng/L) | -15.4 | -43.4, 12.7 | 0.284 |
| GFAP (ng/mL) | -2.9 | -6.5, 0.682 | 0.113 |
| NSE (ng/mL) | 2.8 | -7.0, 12.6 | 0.572 |
| MMP-9 (ng/mL) | 2.5 | -203, 208 | 0.981 |

No group-time interaction terms were significant for any of the above models. Data presented reflect the desaturation group compared to the non-desaturation group control group (baseline) for the generalized estimating equation models. GFAP = glial fibrillary acid protein; NSE = neuron specific enolase; MMP-9 = matrix metalloproteinase-9.
