# Supplementary material for: Cerebrovascular Disease and Perioperative Neurologic Vulnerability: A Prospective Cohort Study
Source: Front Neurol. 2019 May 28;10:560. doi: 10.3389/fneur.2019.00560 (PMC6558425; doi:10.3389/fneur.2019.00560)
Supplement: Supplementary file 6 [file Image_1.pdf]

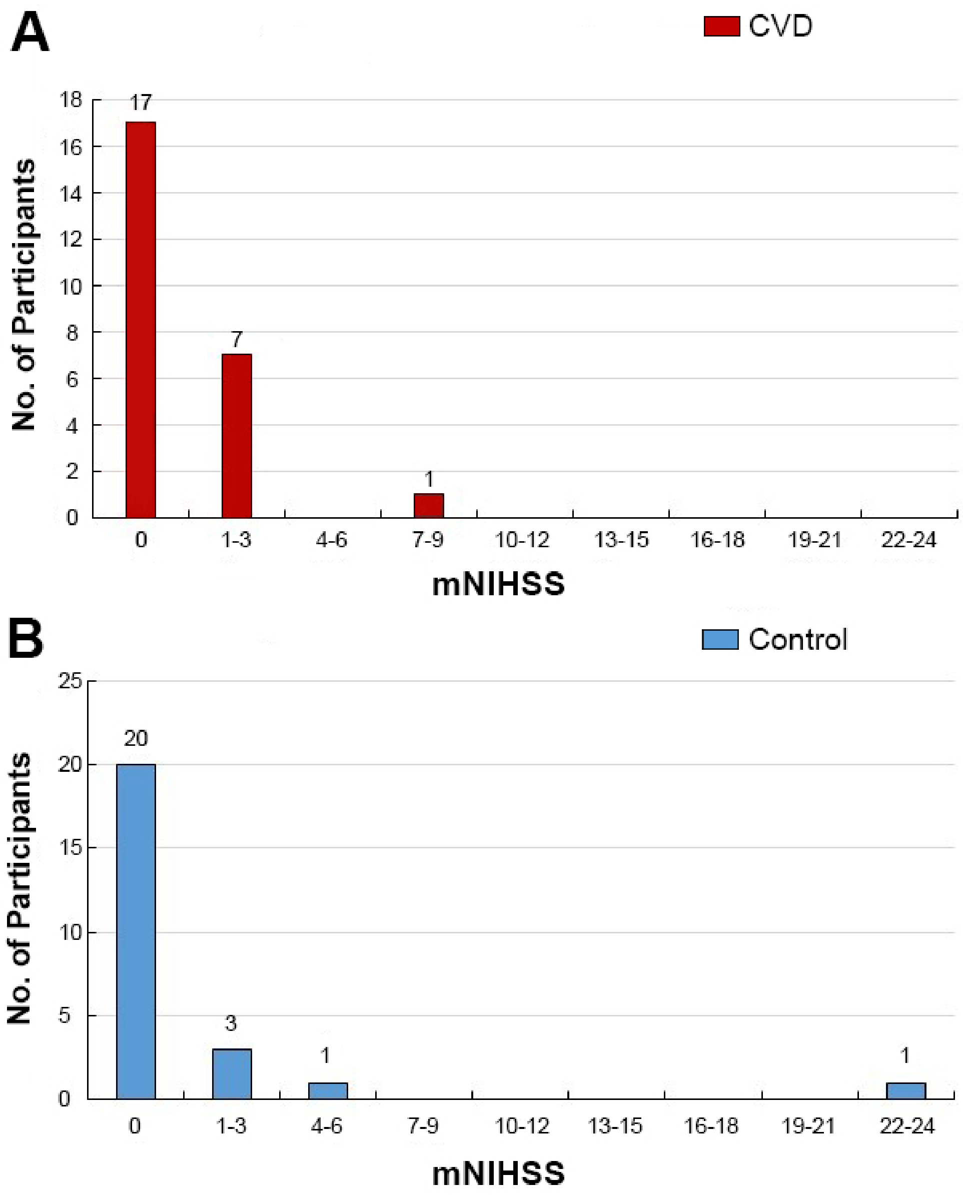

Supplementary Figure 1. Proportion of participants with increased modified NIH Stroke Scale (mNIHSS) scores for (A) CVD (cerebrovascular disease) and (B) control groups. Exams were performed once daily for the first three postoperative days, and any increase from baseline during this timeframe was reported. There was no significant overall difference between the groups (P=0.328).
